# Association of Maternal Dietary Patterns during Gestation and Offspring Neurodevelopment

**DOI:** 10.3390/nu14040730

**Published:** 2022-02-09

**Authors:** Siyuan Lv, Rui Qin, Yangqian Jiang, Hong Lv, Qun Lu, Shiyao Tao, Lei Huang, Cong Liu, Xin Xu, Qingru Wang, Mei Li, Zhi Li, Ye Ding, Ci Song, Tao Jiang, Hongxia Ma, Guangfu Jin, Yankai Xia, Zhixu Wang, Shanshan Geng, Jiangbo Du, Yuan Lin, Zhibin Hu

**Affiliations:** 1 State Key Laboratory of Reproductive Medicine, Nanjing Medical University, Nanjing 211166, China; lvsiyuan_njmu@163.com (S.L.); qinrui@njmu.edu.cn (R.Q.); jiangyangqian23@163.com (Y.J.); lvhong1203@njmu.edu.cn (H.L.); luqun@njmu.edu.cn (Q.L.); taoshiyao_njmu@163.com (S.T.); leihuang@njmu.edu.cn (L.H.); lc1454732687@126.com (C.L.); xx3411251996@163.com (X.X.); wangqingru_0228@163.com (Q.W.); limei_njmu@163.com (M.L.); lizhi@njmu.edu.cn (Z.L.); hongxiama@njmu.edu.cn (H.M.); guangfujin@njmu.edu.cn (G.J.); yankaixia@njmu.edu.cn (Y.X.); zhibin_hu@njmu.edu.cn (Z.H.); 2 Department of Toxicology and Nutritional Science, School of Public Health, Nanjing Medical University, Nanjing 211166, China; 3 Department of Epidemiology, Center for Global Health, School of Public Health, Nanjing Medical University, Nanjing 211166, China; songci@njmu.edu.cn; 4 Suzhou Municipal Hospital, Gusu School, Nanjing Medical University, Suzhou 215002, China; 5 Department of Maternal, Child and Adolescent Health, Center for Global Health, School of Public Health, Nanjing Medical University, Nanjing 211166, China; dingye@njmu.edu.cn (Y.D.); zxwang@njmu.edu.cn (Z.W.); 6 Key Laboratory of Modern Toxicology of Ministry of Education, School of Public Health, Nanjing Medical University, Nanjing 211166, China; 7 Department of Biostatistics, School of Public Health, Nanjing Medical University, Nanjing 211166, China; tao.chiang0923@njmu.edu.cn

**Keywords:** birth cohort, prospective study, maternal dietary pattern, neurodevelopment

## Abstract

The health effects of diet are long term and persistent. Few cohort studies have investigated the influence of maternal dietary patterns during different gestational periods on offspring’s health outcomes. This study investigated the associations between maternal dietary patterns in the mid- and late-gestation and infant’s neurodevelopment at 1 year of age in the Jiangsu Birth Cohort (JBC) Study. A total of 1178 mother–child pairs were available for analysis. A semiquantitative food frequency questionnaire (FFQ) was used to investigate dietary intake at 22–26 and 30–34 gestational weeks (GWs). Neurodevelopment of children aged 1 year old was assessed using Bayley-Ⅲ Screening Test. Principal component analysis (PCA) and Poisson regression were used to extract dietary patterns and to investigate the association between dietary patterns and infant neurodevelopment. After adjusting for potential confounders, the maternal ‘Aquatic products, Fresh vegetables and Homonemeae’ pattern in the second trimester was associated with a lower risk of being non-competent in cognitive and gross motor development, respectively (cognition: aRR = 0.84; 95% CI 0.74–0.94; gross motor: aRR = 0.80; 95% CI 0.71–0.91), and the similar pattern, ‘Aquatic products and Homonemeae’, in the third trimester also showed significant association with decreased risk of failing age-appreciate cognitive and receptive communication development (cognition: aRR = 0.89; 95% CI 0.80–0.98; receptive communication: aRR = 0.91; 95% CI 0.84–0.99). Notably, adherence to the dietary pattern with relatively high aquatic and homonemeae products in both trimesters demonstrated remarkable protective effects on child neurodevelopment with the risk of being non-competent in cognitive and gross motor development decreasing by 59% (95% CI 0.21–0.79) and 63% (95% CI 0.18–0.77), respectively. Our findings suggested that adherence to the ‘Aquatic products and Homonemeae’ dietary pattern during pregnancy may have optimal effects on offspring’s neurodevelopment.

## 1. Introduction

Early neurodevelopmental delays have negative impacts on children’s daily life and school performance, which has become a pressing public health concern [1,2]. Fetal neurodevelopment begins approximately 22 days after conception and develops rapidly in the second and third trimesters [3]. Existing evidence has demonstrated the prominent benefits on fetal neurodevelopment from maternal dietary patterns during pregnancy in a reasonable amount of food and nutrients [4,5,6,7,8,9,10]. In contrast, the adverse influences of an unhealthy diet during pregnancy on the neurodevelopment of offspring remains after birth and even for long term. For instance, a randomized-controlled trial demonstrated that the maternal diet of two servings of cod per week during pregnancy might have adverse effects on infants’ neurodevelopment [11]. A prospective birth cohort study found that maternal intake of excess folic acid supplements during pregnancy was associated with adverse psychomotor development in children after the first year of life [12]. The potential mechanisms include the alteration of epigenetics of fetal genome and the regulation of fetal brain-derived neurotrophic factor (BDNF) levels by maternal nutrition [13,14,15].

Indeed, considering the complexity of dietary status during pregnancy, individual foods and nutrients cannot represent the overall nutritional status. Dietary patterns, proven to be simple, useful, comprehensive and complementary, have been well-accepted and increasingly used by nutritionists to assess the overall dietary status [16,17]. Epidemiological data on dietary patterns during pregnancy and neurodevelopment in offspring after birth are currently sparse. One study conducted within the Avon Longitudinal Study of Parents and Children (ALSPAC) cohort suggested that children born to mothers who were prone to the ‘Meat and Potatoes’ pattern and the ‘White bread and Coffee’ pattern in the third trimester had lower verbal performance and full-scale intelligence quotient (IQ) at school age as compared to those born to mothers whose dietary pattern was prone to ‘Fruit and Vegetables’ [18]. Additionally, the Generation R Study suggested that both low adherence to a Mediterranean diet and high adherence to a Traditionally Dutch diet during the first trimester were associated with an increased risk of child externalizing problems [19]. Given the considerable heterogeneity in dietary patterns and habits between European and Chinese populations, it is of public health significance to assess the effects of Chinese maternal dietary patterns over gestation on offspring neurodevelopment.

Therefore, in the present study, we aimed to determine the main maternal dietary patterns across gestation in a prospective birth cohort in Jiangsu, China, and further to investigate the maternal dietary patterns in relation to infant neurodevelopment at 1 year of age.

## 2. Materials and Methods

### 2.1. Study Population

The present study is a prospective cohort study within the ongoing Jiangsu Birth Cohort (JBC) Study, which recruits and follows up families receiving assisted reproduction or conceiving naturally in the Women’s Hospital of Nanjing Medical University and the Suzhou Affiliated Hospital of Nanjing Medical University to investigate the heterogeneity of assisted versus natural pregnancies in perinatal outcomes and children health, and to systematically assess potential influencing factors for child health and wellbeing. Details of the JBC Study have been published elsewhere [20]. Starting in April 2017, women were asked to complete the semiquantitative food frequency questionnaire (FFQ) in the first (10–14 weeks), second (22–26 weeks), and third (30–34 weeks) trimester of gestation. They were contacted again for children’s follow-up at 42 days, 6 months, and 1 year. The initial study and subsequent follow-ups of the women and their children were approved by the Human Research Ethics Committee of Nanjing Medical University. Written informed consent was obtained from all participants. The ethical approval code for the project is NJMUIRB (2017) 002.

**Figure 1:**
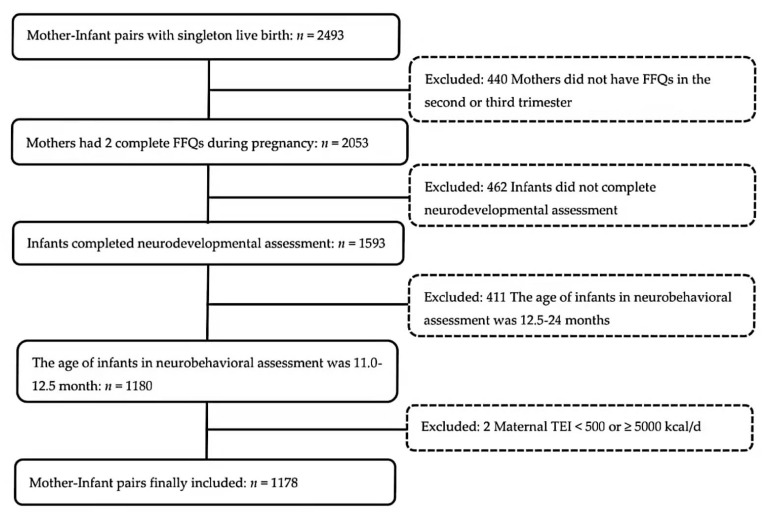
Participants flow chart. (Abbreviations: FFQ = food frequency questionnaire; TEI = total energy intake).

By September 2020, a total of 2493 singleton children in the cohort reached 1 year of age. We excluded children from the present study if (1) their mothers did not complete two FFQs (one in the second trimester, and one in the third trimester) (*n* = 440); (2) they did not complete the Bayley-Ⅲ Screening Test (*n* = 462); (3) they completed the Bayley-Ⅲ Screening Test aged above 12.5 months (*n* = 411); (4) their mothers reported daily energy intake <500 kcal/d or ≥5000 kcal/d (*n* = 2). Finally, a total of 1178 mother–infant pairs were included in the analysis. A detailed inclusion and exclusion flow chart was shown in Figure 1.

### 2.2. Dietary Assessment

Dietary intake information was assessed during the second and the third trimester, using the semiquantitative FFQ. The FFQ was administered by well-trained interviewers with food models and food maps [21] when women received face-to-face follow-ups. In the FFQ, the frequency and amounts of 25 food items were recorded. The validity of the FFQ have been reported in Table S1. The daily nutrient intake was calculated by multiplying the daily frequency of consumption, the amount per serving, and the nutrient per gram, which were obtained through the Chinese Food Composition Table [22]. Additionally, the dietary data used in this paper were converted logarithmically first, and then the residual method [23] was used for energy correction.

### 2.3. Neurodevelopment Evaluation

At the 1-year follow-up, children’s neurodevelopment was assessed by licensed psychologists using the Bayley-Ⅲ Screening Test, accompanied by their primary caregiver. The Bayley-Ⅲ Screening Test is one of the most commonly used screening tools for assessing neurodevelopmental function, for which the effectiveness and reliability has been proved [24]. The Bayley-Ⅲ Screening Test consists of five domains: cognition, expressive communication, receptive communication, fine motor, and gross motor. The children who received the test were categorized as ‘at risk’, ‘emerging’ or ‘competent’ according to the cutoff points for specific age [25]. In this study, we classified ‘at risk’ and ‘emerging’ as ‘non-competent’. Additionally, in order to ensure the validity and reliability of the neurodevelopment evaluation, the JBC Study have taken a series of measures. Specifically, all the psychologists have undergone rigorous training by one appointed developmental neuropsychologist. After the approval of the guardians, the whole process of assessments was filmed. All psychologists were not aware of the dietary status of mothers.

### 2.4. Potential Confounders

All procedures were conducted according to standardized protocols. Maternal and child socio-demographic and lifestyle characteristics from early pregnancy to 1 year after birth were obtained via face-to-face or telephone questionnaires and medical records. Mode of conception (spontaneous pregnancy (SP)/assisted reproductive technology (ARTP)), maternal age, maternal pre-pregnancy BMI, parity (primipara/multipara), and folic acid supplements intake (mg/day) were collected at recruitment. We obtained information on maternal hypertension (chronic or pregnancy-induced hypertension, yes/no), maternal diabetes (pre-pregnancy or gestational diabetes, yes/no), gestational week at delivery, and infant sex (male/female) from electronic medical records (EMR). At the 1-year follow-up, we collected details on the duration of breastfeeding (<6/6–12 months) and child age when receiving the Bayley assessment.

### 2.5. Statistical Analysis

Dietary patterns were extracted by principal component analysis (PCA), which turned a large number of related variables into a small set of unrelated variables and maximized the explained variance. The foods listed in the food frequency questionnaire were first grouped into 16 food groups based on the similarity of the nutritional content of food. The intake of these food groups (g/day) were log transformed and then were adjusted using the residual method for total energy intake [23]. According to the scree plot and interpretability, we selected the eigenvalue of >1.3 criterion and determined the number of components [26]. We named the selected components based on factor loadings above 0.5 (Table S2), and the factor scores for each pattern and for each individual were derived from the component’s factor loadings for the food groups and the calculated food intake. Finally, four dietary patterns were extracted during the second and third trimesters, respectively.

After adjusting for potential confounders, Poisson regression analysis was conducted to investigate the association between different dietary patterns scores and categorical outcomes of infant’s neurodevelopment. The intraclass correlation coefficient (ICC) and 95% CI were calculated by dietary pattern scores to assess temporal variability of dietary patterns during pregnancy. Additionally, continuous variables were expressed in terms of mean and standard deviation, and classified variables were expressed in terms of frequency (*n*) and percentage (%). All hypothesis testing was conducted assuming a 0.05 significance level and a two-sided alternative hypothesis. All statistical analyses were performed using R software (Version 3.6.1, R Foundation for Statistic Computing, Vienna, Austria. URL https://www.R-project.org/ (accessed on 30 January 2022)).

## 3. Results

### 3.1. Basic Characteristics and Neurodevelopmental Assessment

Descriptive characteristics of 1178 mother–infant pairs were presented in Table 1. In our study population, the mean age of mothers at delivery was 31 years old. One in five women were overweight or obese before pregnancy, and approximately 30% of them were complicated with chronic diabetes or gestational diabetes during gestation. Given that the cohort was originally designed to investigate the heterogeneity of assisted versus natural pregnancies in perinatal outcomes and child health, we had forty percent mothers conceived after assisted reproduction (*n* = 471) included in the study. Less than 1% of women smoked or drank during pregnancy. With regard to the infants included, 3.8% (*n* = 45) were born preterm and 2.3% (*n* = 27) had a low birth weight under 2500 g. The prevalence of non-competent neurodevelopment among 1178 infants ranged from 4.1% (fine motor) to 17.5% (receptive language). Additionally, we compared the characteristics of mothers who completed the FFQ in both the second and the third trimesters with those mothers who did not complete the FFQs as requested. As shown in Table S3, women who did not complete the FFQs were more likely to be those who conceived after ART, and delivered preterm and LBW babies. No differences were observed in maternal age, pre-pregnancy BMI, parity and prevalence of diabetes or hypertension during pregnancy.

### 3.2. Maternal Dietary Patterns across Gestation

In the second and the third trimester, four dietary patterns were extracted, respectively. The ‘Aquatic products and Homonemeae’ and ‘Nut’ patterns were observed in both trimesters. The ‘Aquatic products and Homonemeae’ pattern, which was characterized by high consumption of high dietary fiber and high-quality protein, explained 9.7% and 10.0% of the variation in the dietary data in the second and third trimesters, respectively. The ‘Nut’ pattern, mainly associated with consumption of nut, explained 8.6% and 8.7% of the variation in the second and third trimesters, respectively. In addition, the ‘Haslet, Beans, Shells and Molluscs’ pattern and ‘Sweets’ pattern were determined in the second trimester, and ‘Pome, Berry and Melon fruits’ pattern and ‘Citrus’ pattern were observed in the third trimester, which reflected the variation in the dietary pattern in the mid and late gestation. Detailed factor loadings for the food groups with different dietary patterns were shown in Table S2. In the evaluation of temporal variability of dietary across pregnancy, the ‘Aquatic products and Homonemeae’ dietary pattern showed high consistency (ICC > 0.40) from mid- to late gestation in our study population, while the ‘Nut’ pattern fluctuated significantly (Table S4).

### 3.3. Maternal Dietary Patterns and Neurodevelopment in Infants

**Figure 2:**
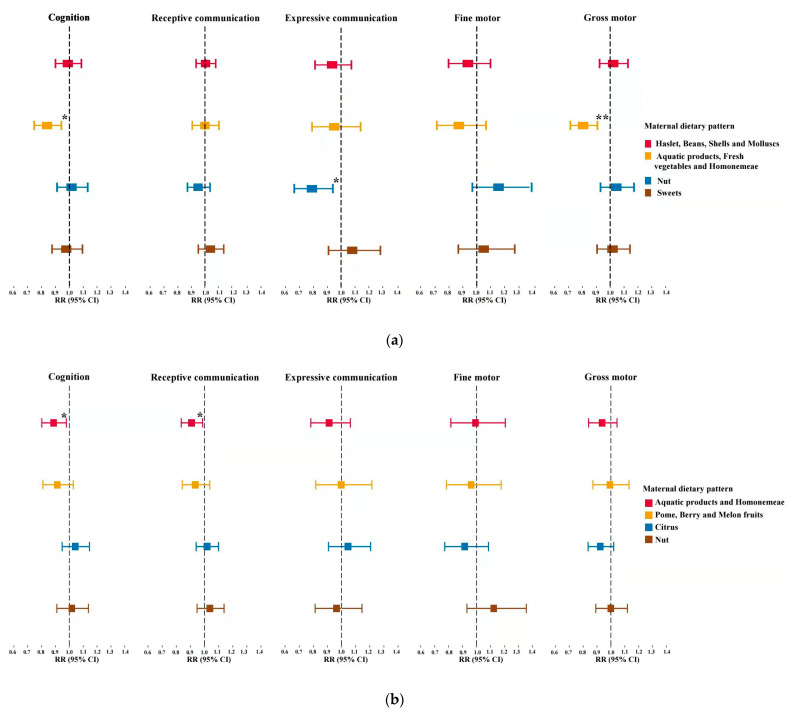
Adjusted associations of dietary patterns in the second and third trimesters with categorical outcomes of neurodevelopment in infants, respectively. Analyses were adjusted for mode of conception, maternal age, maternal pre-pregnancy body mass index (BMI), parity, hypertension, diabetes, gestational week at delivery, infant sex, duration of breastfeeding, principal component score of maternal dietary intake, folic acid supplements. * *p* < 0.05, ** *p* < 0.001. (**a**), Second trimester; (**b**), Third trimester. Abbreviations: RR = Risk ratio; 95% CI = 95% confidence intervals.

Among the 1178 infants, the multivariable associations of maternal dietary patterns in the second and the third trimester with the risk of nonoptimal neurodevelopment in infants were presented in Figure 2 and Table S5. A higher adherence score of ‘Aquatic products, Fresh vegetables and Homonemeae’ food consumption in the second trimester was significantly associated with decreased risk in infants being non-competent in cognitive development (aRR = 0.84; 95% CI 0.75–0.93) and in gross motor development (aRR = 0.82; 95% CI 0.73–0.91) after adjusting for conventional covariates including mode of conception, maternal age, maternal pre-pregnancy BMI, parity, gestational week at delivery, hypertension or diabetes during pregnancy, infant sex, and duration of breastfeeding (Model 1). After the further adjustments for folic acid supplementation and the scores for the rest dietary patterns (Model 2), the associations remained steady. Notably, similar maternal dietary pattern, ‘Aquatic products and Homonemeae’ pattern in the third trimester, also demonstrated optimal effects on infant’s neurodevelopment with higher adherence score being associated with lower risk of nonoptimal cognitive development (Model 2: aRR = 0.89; 95% CI 0.80–0.98) and receptive communication development (Model 2: aRR = 0.91; 95% CI 0.84–0.99). In addition, the adherence score of the ‘Nut’ pattern in the second trimester was observed associated with decreased risk of infants being non-competent in expressive communication (Model 2: aRR = 0.79; 95% CI 0.66–0.94), while the association diminished in the third trimester. We did not observe the rest dietary patterns in mid- and later gestation associated with infant’s neurodevelopment in any of the five domains.

### 3.4. Adherence to ‘Aquatic Products and Homonemeae’ Pattern and Infant Neurodevelopment

To assess the adherence to the ‘Aquatic products and Homonemeae’ pattern from mid- to late pregnancy in relation to neurodevelopment in infants, we categorized participants into tertiles according to their scores of dietary patterns in each trimester. As compared to the infants whose mothers at the lowest tertile of ‘Aquatic products and Homonemeae’ pattern in both trimesters, those whose mothers at the highest tertile in both trimesters demonstrated 59% (Model 2: aRR = 0.41; 95% CI 0.21–0.79; *p* = 0.008) and 63% (Model 2: aRR = 0.37; 95% CI 0.18–0.77; *p* = 0.008) decreased risk of being non-competent in cognitive and gross motor development after adjusting for potential confounders, respectively (Table 2).

## 4. Discussion

It is well known that children’s neurodevelopment is affected by various factors such as mother’s lifestyle [27], environmental pollution [28], food contamination with chemical compounds [29], and pregnancy leisure physical activity [30]. Notably, maternal dietary intake has been extensively studied due to its greater controllability. This prospective birth cohort study conducted in Jiangsu province, China, investigated maternal dietary patterns across mid- to late gestation in relation to infant neurodevelopment. Our results demonstrated that the maternal dietary pattern of ‘Aquatic products and Homonemeae’ in the second and the third trimester was associated with a lower risk of being non-competent in cognitive development in infants at 1 year of age. Particularly, adherence to this pattern in both trimesters reduced the risk by 59% and 63% for nonoptimal development in cognition and gross motor skills, respectively.

The use of dietary patterns to consider the interactions of various foods and nutrients simultaneously is becoming increasingly common. Western dietary patterns, such as the ‘DASH dietary pattern’ and the ‘Mediterranean-type diet’, have been linked to lower risk of maternal and infant diseases [31,32], while the ‘Western dietary pattern’ can increase the risk of adverse health outcomes [33]. The Chinese dietary patterns extracted by a data-driven method reflected the local food consumption habits well. The Guangzhou Cohort Study demonstrated that a maternal diet with lots of milk and few vegetables during pregnancy may be associated with an increased risk of preterm delivery [34]. While no previous study has examined the relation between maternal dietary patterns in China during pregnancy and subsequent children neuropsychological function.

The neuroprotective effects of the ‘Aquatic, Fresh vegetables and Homonemeae’ pattern may result from the biological properties of the nutrients contained. Aquatic products are good sources of high-quality protein, omega-3 (ω-3) fatty acids and minerals such as iodine. Previous studies have demonstrated that omega-3 polyunsaturated fatty acid (ω-3 PUFA) may play key roles in neural development. The ALSPAC cohort study reported that the encouragement of maternal seafood consumption during the third trimester may benefit the neurodevelopment of children aged 6 months to 8 years [35]. In addition, several meta-analyses showed that children with attention deficit hyperactivity disorder (ADHD) have lower blood levels of ω-3 PUFA [36,37], and two observational studies suggested that higher dietary intake of fish or ω-3 PUFA can reduce the risk of psychosis [37,38]. Existing animal experiments showed that a diet rich in omega-3 reduced inflammation in the hypothalamus in rats [39]. In addition, maternal iodine has been associated with children neurodevelopment. For instance, the Southampton Women cohort reported that maternal urinary iodine concentration was positively correlated with cognitive function in children aged 6–7 years [40]. It has been demonstrated that iodine deficiency in mothers leads to insufficient thyroid hormone production, which further affects neuronal proliferation and migration in the cortex and hippocampus of their offspring [41,42]. In an animal experiment, maternal mild iodine deficiency promoted developmental hypothyroxinaemia, which further led to highly retarding the ability of offspring’s hippocampal axonal growth in rats [43]. Homonemeae, with well-balanced essential amino acids and rich iodine [44], has been known for its neuroprotective effects. Furthermore, evidence from cell experiments suggested that dimethylsulfoniopropionate (DMSP) produced by homonemeae could strengthen antioxidant defense in mammalian nerve cells [45]. In addition, fresh vegetables are rich in folate, vitamin C, beta carotene (provitamin A), and other nutrients. More fresh vegetables in a mother’s diet have been suggested in relation to better neural development in offspring [18,46]. Given the difficulty of collecting dietary data repeatedly, the combined effects of maternal dietary over gestation are less studied in prior studies. A notable finding of the present study is the remarkable protective effects we observed of adherence to the ‘Aquatic products, Fresh vegetables and Homonemeae’ dietary pattern on child neurodevelopment, which indicates the cumulative effect of dietary nutrients on neural development.

The advantages of this study should be elaborated. First, the use of dietary patterns captured the overall quality of the diet and were good proxy for dietary status over time [47]. In addition, abundant data on maternal and child characteristics were collected from a prospective and longitudinal cohort study to control potential confounding factors. Finally, a range of approaches were adopted to guarantee the validity and reliability of outcome measures. The study also has some limitations. First, recall bias may be caused by FFQ because it requires participants to recall their food intake over a period of time. However, we compared the estimated daily intake using FFQ with the mean of 3-day 24 h dietary recall (24HR) and proved that FFQ had comparable validity in a pre-survey. Second, residual confounding may occur due to other unmeasured confounding factors. In our analysis, factors related to fetal neurodevelopment such as maternal folic acid supplements and other dietary patterns, were considered as much as possible. Third, though with available maternal dietary data, we did not determine and assess the dietary pattern in the first trimester. A large number of women experience nausea and vomiting in their early pregnancy and thus the dietary pattern might not be well correlated to actual food and nutrient consumption.

## 5. Conclusions

In the prospective and longitudinal birth cohort study, our results suggested the cumulative effects of maternal diet on infant neurodevelopment. These findings highlighted the optimal effects of adherence to the ‘Aquatic products and Homonemeae’ dietary pattern during pregnancy and might provide add-on evidence to nutrition counseling for pregnant women.

## Figures and Tables

**Table 1:** Descriptive characteristics of baseline demographic and lifestyle factors of 1178 mother–infant pairs *.

	Overall
Maternal age at delivery (years)	31.01 (3.92)
Maternal pre-pregnancy BMI (kg/m^2^)	21.68 (2.98)
<18.5	127 (10.8)
18.5–23.9	807 (68.5)
24–27.9	189 (16.0)
≥28	46 (3.9)
Diabetes ^a^	339 (28.8)
Hypertension ^b^	60 (5.1)
Mode of conception	
SP	707 (60.0)
ARTP	471 (40.0)
Primipara	915 (77.7)
Tobacco use during pregnancy	1 (0.1)
Alcohol intake during pregnancy	5 (0.4)
Preterm birth	45 (3.8)
Infant sex	
Male	612 (52.0)
Female	566 (48.0)
LBW (<2500 g) ^c^	27 (2.3)
Duration of breastfeeding, months	
<6	406 (34.5)
6–12	754 (64.0)
Bayley-Ⅲ screening test scale	
Non-competent in cognition	142 (12.1)
Non-competent in receptive communication	206 (17.5)
Non-competent in expressive communication	62 (5.3)
Non-competent in fine motor skills	48 (4.1)
Non-competent in gross motor skills	131 (11.1)

* Data are presented as mean (SD) or *n* (%). Abbreviations: BMI = body mass index; SP = spontaneous pregnancy; ARTP = assisted reproductive technology pregnancy. ^a^ Diabetes includes chronic and gestational diabetes. ^b^ Hypertension includes chronic, gestational and pre-eclampsia. ^c^ Low birth weight.

**Table 2:** Multivariable associations of adherence to the ‘Aquatic products and Homonemeae’ pattern in the second and third trimesters and categorical outcomes of neurodevelopment in infants.

		Cognition		Receptive Communication		Expressive Communication		Fine Motor		Gross Motor	
		RR (95% CI)	*p*	RR (95% CI)	*p*	RR (95% CI)	*p*	RR (95% CI)	*p*	RR (95% CI)	*p*
Model 1											
Lowest tertile in both trimesters	*n* = 196	Ref		Ref		Ref		Ref		Ref	
Highest tertile in both trimesters	*n* = 180	0.40 (0.21, 0.78)	0.007	0.75 (0.47, 1.20)	0.232	0.84 (0.37, 1.93)	0.682	0.45 (0.16, 1.31)	0.142	0.37 (0.18, 0.76)	0.007
Model 2											
Lowest tertile in both trimesters	*n* = 196	Ref		Ref		Ref		Ref		Ref	
Highest tertile in both trimesters	*n* = 180	0.41 (0.21, 0.79)	0.008	0.77 (0.48, 1.23)	0.266	0.86 (0.38, 1.98)	0.724	0.46 (0.16, 1.33)	0.150	0.37 (0.18, 0.77)	0.008

Model 1: adjusted for mode of conception, maternal age, maternal pre-pregnancy BMI, parity, hypertension, diabetes, gestational week at delivery, infant sex, duration of breastfeeding. Model 2: adjusted for mode of conception, maternal age, maternal pre-pregnancy BMI, parity, hypertension, diabetes, gestational week at delivery, infant sex, duration of breastfeeding, folic acid supplements. Statistically significant differences are highlighted in bold. Abbreviations: Ref = Reference group, which is the group of mothers with the lowest tertile of dietary pattern scores in both trimesters.

## Data Availability

All data analyzed or generated during this study are available from the corresponding authors on reasonable request.

## Supplementary Materials

The following supporting information can be downloaded at: https://www.mdpi.com/article/10.3390/nu14040730/s1, Table S1: Pearson correlation coefficients between FFQ and 3-day 24 h completed by 141 pregnant women in the second trimester; Table S2: dietary patterns identified by PCA and factor loadings of food groups/items included in each dietary pattern for the pregnant woman in her second and third trimesters (*n* = 1178); Table S3: comparison between characteristics of eligible mothers who completed the FFQ at both trimesters and those who did not complete as requested; Table S4: distribution of similar dietary pattern scores across gestation; Table S5: multivariable associations between maternal dietary patterns in the second and third trimesters and categorical outcomes of neurodevelopment in infants.
