# Supplementary material for: Association of Maternal Dietary Patterns during Gestation and Offspring Neurodevelopment
Source: Nutrients. 2022 Feb 9;14(4):730. doi: 10.3390/nu14040730 (PMC8878236; doi:10.3390/nu14040730)
Supplement: Supplementary file 1 [file nutrients-14-00730-s001.zip › nutrients-1563008-supplementary.pdf]

Supplemental Table S1. Pearson correlation coefficients between FFQ and 3–day 24HR completed by 141 pregnant women in the second trimester.

Daily food intakes (g/d)	FFQ	3–day 24HR	TE adjusted and de-attenuated†	*p*††
Refined cereals	4.48 (3.99, 4.66)	4.29 (3.94, 4.68)	0.21	0.011
Eggs	4.60 (3.84, 4.96)	4.14 (3.75, 4.42)	0.40	<0.001
Meat	5.13 (4.33, 5.45)	4.38 (3.90, 4.71)	0.40	<0.001
Beans	3.43 (2.00, 4.34)	3.30 (1.28, 4.46)	0.32	<0.001
Sweets	2.80 (1.35, 3.54)	2.93 (0.74, 3.65)	0.39	<0.001
Citrus	2.45 (0.20, 3.52)	2.18 (0.04, 4.39)	0.51	<0.001
Nut	2.35 (0.84, 3.20)	1.37 (0.15, 2.95)	0.42	<0.001
Homonemeae	2.48 (1.37, 2.97)	1.28 (0.06, 2.79)	0.22	0.007
Dairy	5.70 (5.14, 6.06)	5.10 (4.47, 5.56)	0.59	<0.001
Fried food	0.53 (0.10, 1.94)	0.05 (−0.03, 0.15)	0.14	0.092
Tubers and Whole grains	3.49 (2.88, 4.15)	4.65 (4.17, 5.13)	0.16	0.056
Haslet	2.04 (0.62, 2.78)	0.06 (−0.03, 1.96)	0.27	0.001
Shells and Molluscs	0.10 (−0.01, 0.68)	0.09 (−0.12, 0.22)	0.23	0.006
Aquatic products	3.76 (3.30, 4.23)	3.87 (3.15, 4.56)	0.21	0.011
Fresh vegetables	4.42 (4.09, 4.65)	4.87 (4.45, 5.32)	0.20	0.016
Pome, Berry and Melon fruits	5.50 (5.22, 5.86)	5.45 (5.19, 5.85)	0.33	<0.001

†Pearson correlation coefficients between average intakes of energy and nutrients from FFQ and those obtained from 3-day 24HR, respectively.

††P Value of Pearson correlation coefficients after energy adjustment and de attenuation.

FFQ, food frequency questionnaire; 24HR, 24-hour dietary recall; TE, total energy.

Supplemental Table S2. Dietary patterns identified by PCA and factor loadings of food groups/items* included in each dietary pattern for the pregnant woman in her second and third trimesters (n=1,178).

	Dietary Pattern							
	the second trimester				the third trimester			
	‘Haslet, Beans, Shells and Molluscs’	‘Aquatic products, Fresh vegetables and Homonemeae’	‘Nut’	'‘Sweets’	‘Aquatic products and Homonemeae’	‘Pome, Berry and Melon fruits’	‘Citrus’	‘Nut’
Food items								
Refined cereals			−0.544					
Eggs								
Meat				−0.629				−0.801
Beans	0.532							
Sweets				0.652				
Citrus							0.629	
Nut			0.605					0.643
Homonemeae		0.513			0.671			
Dairy								
Fried food								
Tubers and Whole grains						−0.522		
Haslet	0.589							
Shells and Molluscs	0.561							
Aquatic products		0.686			0.675			
Fresh vegetables		0.705					−0.524	
Pome, Berry and Melon fruits						0.683		
Variance explained (%)	9.800	9.700	8.600	8.600	10.000	9.900	9.500	8.700

Abbreviations: PCA, principal component analysis.

*Only food groups/items with factor loadings >0.50 or <−0.5 were retained for each factor.

Supplemental Table S3. Comparison between characteristics of eligible mothers who completed the FFQ at both trimesters and those who did not complete as requested*.

		Included (n = 1178)	Not included (n = 1257)	p
**Maternal characteristics**				
Maternal age at delivery (years)		31.01 (3.92)	31.05 (3.90)	0.845
Maternal pre-pregnancy BMI (kg/m2)				
	<18.5	127 (10.8)	164 (13.0)	0.480
	18.5–23.9	807 (68.5)	832 (66.2)	
	24−27.9	189 (16.0)	197 (15.7)	
	≥28	46 (3.9)	52 (4.1)	
Diabetes [a]		339 (28.8)	308 (25.3)	0.059
Hypertension [b]		60 (5.1)	58 (4.8)	0.793
Mode of conception				
	SP	707 (60.0)	689 (54.8)	**0.011**
	ARTP	471 (40.0)	568 (45.2)	
Primipara		915 (77.7)	975 (77.6)	0.626
**Infants characteristics**				
Preterm birth		45 (3.8)	91 (7.2)	**<0.001**
Infant sex				
	Male	612 (52.0)	675 (53.7)	0.060
	Female	566 (48.0)	577 (45.9)	
LBW (<2500 g) [c]		27 (2.3)	64 (5.1)	**0.001**
Duration of breastfeeding, months				
	<6	406 (34.5)	327 (26.0)	**<0.001**
	6–12	754 (64.0)	512 (40.7)	

* Data are presented as mean (SD) or n (%). *P* values obtained by independent t-test or chi-square analysis.

Abbreviations: BMI = body mass index;

SP = spontaneous pregnancy;

ARTP = assisted reproductive technology pregnancy.

[a] Diabetes includes chronic and gestational diabetes.

[b] Hypertension includes chronic, gestational and pre-eclampsia.

[c] Low birth weight.

Supplemental Table S4. Distribution of similar dietary pattern scores across gestation.

Dietary Pattern	Median (IQR)		ICC (95% CI)
	Second trimester	Third trimester	
'Aquatic products, Fresh vegetables and Homonemeae'	9.27 (8.33, 10.01)	10.71 (9.66, 11.65)	0.461 (0.407, 0.510)
'Nut'	−1.15 (−2.11, −0.14)	−1.01 (−2.17, −0.05)	0.330 (0.260, 0.390)

IQR, interquartile range; ICC, intraclass correlation coefficient.

Supplemental Table S5. Multivariable associations between maternal dietary patterns in the second and third trimesters and categorical outcomes of neurodevelopment in infants.

	Cognition		Receptive communication		Expressive communication		Fine motor		Gross motor	
	**RR （95%CI）**	***p***	**RR （95%CI）**	***p***	**RR （95%CI）**	***p***	**RR （95%CI）**	***p***	**RR （95%CI）**	***p***
Second trimester										
‘Haslet, Beans, Shells and Molluscs’										
Model1	0.95 (0.87, 1.04)	0.294	1.01 (0.94, 1.09)	0.758	0.95 (0.82, 1.08)	0.422	0.91 (0.78, 1.06)	0.216	0.99 (0.90, 1.08)	0.759
Model2	0.99 (0.90, 1.09)	0.811	1.00 (0.93, 1.09)	0.912	0.93 (0.81, 1.08)	0.338	0.94 (0.80, 1.10)	0.416	1.02 (0.92, 1.13)	0.684
‘Aquatic products, Fresh vegetables and Homonemeae’										
Model1	**0.84 (0.75, 0.93)**	**0.001**	0.98 (0.89, 1.08)	0.669	0.86 (0.73, 1.02)	0.079	0.88 (0.72, 1.07)	0.197	**0.82 (0.73, 0.91)**	**<0.001**
Model2	**0.84 (0.74, 0.94)**	**0.003**	1.00 (0.90, 1.11)	0.993	0.95 (0.79, 1.14)	0.581	0.87 (0.71, 1.07)	0.180	**0.80 (0.71, 0.91)**	**<0.001**
‘Nut’										
Model1	0.98 (0.88, 1.09)	0.683	0.94 (0.86, 1.03)	0.201	**0.78 (0.66, 0.92)**	**0.004**	1.13 (0.95, 1.35)	0.167	0.99 (0.88, 1.11)	0.882
Model2	1.02 (0.91, 1.13)	0.781	0.95 (0.86, 1.04)	0.253	**0.79 (0.66, 0.94)**	**0.008**	1.16 (0.97, 1.38)	0.107	1.04 (0.93, 1.17)	0.458
‘Sweets’										
Model1	1.00 (0.89, 1.12)	0.970	1.05 (0.96, 1.15)	0.311	1.09 (0.91, 1.29)	0.356	1.04 (0.85, 1.26)	0.706	1.05 (0.94, 1.19)	0.384
Model2	0.98 (0.87, 1.10)	0.700	1.04 (0.95, 1.15)	0.386	1.08 (0.91, 1.29)	0.376	1.05 (0.87, 1.27)	0.622	1.02 (0.91, 1.14)	0.770
Third trimester										

'Aquatic products and Homonemeae'										
Model1	0.91 (0.82, 1.00)	0.051	**0.92 (0.85, 0.99)**	**0.037**	0.91 (0.79, 1.05)	0.206	0.99 (0.82, 1.18)	0.886	0.92 (0.83, 1.02)	0.115
Model2	**0.89 (0.80, 0.98)**	**0.016**	**0.91 (0.84, 0.99)**	**0.022**	0.91 (0.78, 1.06)	0.236	0.99 (0.82, 1.21)	0.934	0.94 (0.84, 1.04)	0.235
'Pome, Berry and Melon fruits'										
Model1	0.96 (0.86, 1.08)	0.536	0.99 (0.89, 1.09)	0.826	1.06 (0.88, 1.28)	0.542	0.96 (0.80, 1.16)	0.687	1.02 (0.90, 1.16)	0.779
Model2	0.91 (0.81, 1.03)	0.131	0.93 (0.84, 1.04)	0.200	1.00 (0.82, 1.22)	0.975	0.96 (0.78, 1.18)	0.695	0.99 (0.87, 1.13)	0.905
'Citrus'										
Model1	1.02 (0.93, 1.12)	0.638	1.00 (0.93, 1.09)	0.905	1.03 (0.90, 1.19)	0.664	0.92 (0.78, 1.08)	0.297	0.91 (0.83, 1.01)	0.070
Model2	1.04 (0.95, 1.15)	0.387	1.02 (0.94, 1.10)	0.670	1.05 (0.91, 1.21)	0.532	0.92 (0.77, 1.09)	0.306	0.92 (0.84, 1.02)	0.116
'Nut'										
Model1	1.02 (0.91, 1.14)	0.750	1.04 (0.94, 1.14)	0.444	0.97 (0.82, 1.15)	0.717	1.11 (0.92, 1.34)	0.277	0.99 (0.88, 1.11)	0.805
Model2	1.02 (0.91, 1.14)	0.758	1.04 (0.95, 1.14)	0.422	0.97 (0.81, 1.15)	0.683	1.12 (0.93, 1.36)	0.223	1.00 (0.89, 1.12)	0.984

Model 1: adjusted for mode of conception, maternal age, maternal pre-pregnancy BMI, parity, hypertension, diabetes, gestational week at delivery, infant sex, duration of breastfeeding.

Model 2: adjusted for mode of conception, maternal age, maternal pre-pregnancy BMI, parity, hypertension, diabetes, gestational week at delivery, infant sex, duration of breastfeeding, principal component score of maternal dietary intake, folic acid supplements.

Abbreviations: BMI = body mass index.

Statistically significant differences are highlighted in bold.
